# The utilization of systematic review evidence in formulating India’s National Health Programme guidelines between 2007 and 2021

**DOI:** 10.1093/heapol/czad008

**Published:** 2023-01-28

**Authors:** Eti Rajwar, Prachi Pundir, Shradha S Parsekar, Anupama D S, Sonia R B D’Souza, Baby S Nayak, Judith Angelitta Noronha, Preethy D’Souza, Sandy Oliver

**Affiliations:** Public Health Evidence South Asia, Prasanna School of Public Health, Manipal Academy of Higher Education, Madhav Nagar, Manipal, Karnataka 576104, India; The George Institute for Global Health, 308, Third Floor, Elegance Tower, Plot No. 8, Jasola District Centre, New Delhi 110025, India; Public Health Evidence South Asia, Prasanna School of Public Health, Manipal Academy of Higher Education, Madhav Nagar, Manipal, Karnataka 576104, India; The George Institute for Global Health, 308, Third Floor, Elegance Tower, Plot No. 8, Jasola District Centre, New Delhi 110025, India; Department of Community Medicine, Kasturba Medical College, Manipal Academy of Higher Education, Madhav Nagar, Manipal, Karnataka 576104, India; Department of Obstetrics and Gynaecological Nursing, Manipal College of Nursing, Manipal Academy of Higher Education, Madhav Nagar, Manipal, Karnataka 576104, India; Department of Obstetrics and Gynaecological Nursing, Manipal College of Nursing, Manipal Academy of Higher Education, Madhav Nagar, Manipal, Karnataka 576104, India; Department of Child Health Nursing, Manipal College of Nursing, Manipal Academy of Higher Education, Madhav Nagar, Manipal, Karnataka 576104, India; Department of Obstetrics and Gynaecological Nursing, Manipal College of Nursing, Manipal Academy of Higher Education, Madhav Nagar, Manipal, Karnataka 576104, India; EPPI-Centre, Social Science Research Unit, UCL Social Research Institute, University College London, 10 Bedford Way, London WC1H 0AL, UK; EPPI-Centre, Social Science Research Unit, UCL Social Research Institute, University College London, 10 Bedford Way, London WC1H 0AL, UK; Africa Centre for Evidence, Faculty of Humanities, University of Johannesburg, PO Box 524, Auckland Park 2006, Johannesburg, South Africa

**Keywords:** Evidence synthesis, national health programmes, policymaking, public health, India, evidence-informed policymaking

## Abstract

Evidence-informed policymaking integrates the best available evidence on programme outcomes to guide decisions at all stages of the policy process and its importance becomes more pronounced in resource-constrained settings. In this paper, we have reviewed the use of systematic review evidence in framing National Health Programme (NHP) guidelines in India. We searched official websites of the different NHPs, linked to the main website of the Ministry of Health and Family Welfare (MoHFW), in December 2020 and January 2021. NHP guideline documents with systematic review evidence were identified and information on the use of this evidence was extracted. We classified the identified systematic review evidence according to its use in the guideline documents and analysed the data to provide information on the different factors and patterns linked to the use of systematic review evidence in these documents. Systematic reviews were mostly visible in guideline documents addressing maternal and newborn health, communicable diseases and immunization. These systematic reviews were cited in the guidelines to justify the need for action, to justify recommendations for action and opportunities for local adaptation, and to highlight implementation challenges and justify implementation strategies. Guideline documents addressing implementation cited systematic reviews about the problems and policy options more often than citing systematic reviews about implementation. Systematic reviews were linked directly to support statements in few guideline documents, and sometimes the reviews were not appropriately cited. Most of the systematic reviews providing information on the nature and scale of the policy problem included Indian data. It was seen that since 2014, India has been increasingly using systematic review evidence for public health policymaking, particularly for some of its high-priority NHPs. This complements the increasing investment in research synthesis centres and procedures to support evidence-informed decision making, demonstrating the continued evolution of India’s evidence policy system.

Key messages

- Over the years, India has demonstrated a visible evolution in the evidence policy system that can be attributed to two key complementary components of the evidence system (1) development of systematic review generation centres, and (2) commitment to evidence-informed policy development.
- The visibility of systematic reviews in Indian policy documents has been growing since 2014 with international support. Systematic reviews are most visible in policies addressing maternal and newborn health, communicable diseases and immunization. These systematic reviews are sometimes linked directly to support statements in policy documents and other times they appear in a list of additional readings towards the end of the document.
- Systematic reviews are cited in Indian public health policy documents to: (1) justify the need for action; (2) identify key components, experiences, views, effects and costs of policy options to justify recommendations for action and highlight opportunities for local adaptation; and (3) highlight challenges and justify implementation strategies.
- Indian data were mostly used in systematic reviews defining the policy problem rather than assessing different policy options emphasizing the need for methodologies that transfer global evidence about policy options to local settings.
- This growing visibility of systematic reviews in policy documents reflects the earlier global enthusiasm for producing systematic reviews to address perinatal care and epidemiology, and the subsequent developments in systematic reviewing of health systems research and social science (e.g. professional education) more broadly.

## Introduction

Evidence-informed policymaking is an approach to policymaking that involves the use of systematic and transparent research evidence (Oxman *et al.*, 2009a,b). Additionally, the use of views and opinions of stakeholders, policymakers, managers, experts and other groups providing information on contextual factors is an important component of this approach (Lomas, 2005). Evidence-informed policymaking provides clarity and gives information on the nature and extent of the problem, involves clear methods to find and assess research evidence available on policy options, and supports implementation (Oxman *et al.*, 2009a,b).

This approach to policymaking often uses the evidence synthesized via ‘systematic reviews’ to answer policy relevant questions (Oxman *et al.*, 2009b). As an evidence synthesis method, systematic reviews collate all the empirical evidence available to answer a pre-specified research question by using explicit, systematic and reproducible methods (Higgins *et al.*, 2022). These reviews are summaries of the available research evidence that can be used to answer questions about ‘what works’, additionally, systematic reviews can be used to provide an insight into the reasons for how and why a strategy works (e.g. a public health intervention) (Lavis *et al.*, 2003). Another important aspect of evidence-informed policy is the translation of this systematically synthesized evidence into policy and practice. Over the years, many theoretical models for translating research evidence/knowledge to action have been introduced. Knowledge to action (KTA) models such as linear models (knowledge-push and demand-pull models), relational models and systems thinking models give information on how the research evidence can be translated to policy action and the different factors responsible for facilitating the same (Best and Holmes, 2010). Generally, the linear KTA models suggest a one-way approach of knowledge translation, where research evidence (generalizable across contexts), packaged as policy briefs, blogs, documentaries, is transferred from research producers to users and vice versa. In contrast, the relational model of KTA focuses on the importance of shared knowledge, building linkages or collaborations between researchers, decision makers and other stakeholders, for the effective translation of research evidence into policy and practice. This model also focuses on the importance of local-context evidence and its use in evidence-informed policy. The systems thinking model builds on the linear and relational model of KTA and visualizes knowledge transfer from a complex system lens—focusing on building collaborations between organizational networks, leadership supporting organizational change, and effective communication between individuals and organizations (Best and Holmes, 2010). The systems thinking model of KTA also focuses on the different components of use of research evidence, such as creating access to specific research evidence and understanding or interpretation of the available evidence, useful for facilitating evidence-informed policy decisions (Langer *et al.*, 2016).

In India, the history of evidence for clinical decisions began in the 1990s when formal training on evidence-based medicine (EBM) was provided to the medical and nursing faculty of India, by the International Clinical Epidemiology Network (INCLEN) (Prasad, 2013). The importance of good processes for making policy decisions (rather than individual clinical decisions) was also recognized, specifically the value of considering relevant up-to-date knowledge and data, using analytical tools and widespread consultation for comparing policy options, followed by swift implementation (Agarwal and Somanathan, 2005). Yet, early adoption of evidence for clinical decisions in India had to overcome clinicians’ lack of awareness, misinformation or misperceptions of the concept, the complexity of a multistep process, and its absence in the medical curriculum (Agarwal *et al.*, 2008). There were similar barriers to developing standard treatment guidelines: limited understanding, time, enthusiasm and local expertise, and developing consensus between specialists and generalists to prepare guidelines; and when applying guidelines there were concerns about maintaining professional autonomy and treating patients as individuals and applying standards consistently across levels of health care (Sharma *et al.*, 2015).

Evidence-informed decision-making was also recommended for policy because of its transparency, inclusiveness and independence, with attention paid to formal priority setting and resource allocation to avoid political or other competing interests (Bhaumik, 2014). Challenges such as the complexity of the healthcare sector, weaker primary health care, large and unregulated private sector, low spending on health and weak political accountability (Patel *et al.*, 2015) are other likely impediments to the evolving health policymaking process in the country.

Nevertheless, evidence-informed policymaking has seen significant progress over the past few decades and, with systematic review methods now available to synthesize various types of studies, including the context of studies, the methodology is better suited to addressing challenging complex policy issues. Over the years, India has understood the importance of shared research knowledge and building linkages between researchers and decision makers, especially in health policy and systems research. As a result, in 2013 the Ministry of Health and Family Welfare (MoHFW) in India advocated the formation of a National Knowledge Platform to (1) enable knowledge sharing among researchers and policymakers, (2) enable capacity building in research, (3) facilitate research knowledge dissemination, and (4) support research initiatives in priority areas (Sriram *et al.*, 2018). India has been working on achieving the above-mentioned objectives and during this journey has witnessed various milestones, such as establishing Cochrane South Asia, the Health Technology Assessment Board and other evidence synthesis centres, prioritizing evidence-informed policy making in the National Health Policy 2017 and creating task forces for adapting international evidence-based standard treatment guidelines to the Indian context (Mehndiratta *et al.*, 2017). Additionally, the National Institution for Transforming India Aayog (previously the Planning Commission of India; also known as NITI Aayog) introduced evidence into its institutionalized strategic planning framework through researchers and policymakers collaborating to create policy-oriented research (Kattumuri, 2015). More information about the evidence informed policymaking (or EBM) milestones in India is given in Table 1.

**Table 1.** Timeline for milestones in evidence-based medicine and organizations working in evidence generation in India

Year/ period	Milestone/organizations	Details
1993	INCLEN programme having a component of evidence-based medicine (EBM)	India became a part of the International Clinical Epidemiology Network (INCLEN) programme (training on EBM was provided).
1995	First formal workshop in EBM	The first formal workshop on EBM was organized as a part of the annual meeting of the Indian Clinical Epidemiology Network (INDIACLEN) in Kodaikanal, Tamil Nadu.
1998	Unsuccessful attempt to establish Cochrane centre	The Cochrane Collaboration was initiated in the UK in 1993. The first attempt at establishing a Cochrane centre/network in India was in 1998, at the All India Institute of Medical Sciences.
2004	First book publication related to EBM	Book on ‘*Fundamentals of Evidence-Based Medicine*’ published by Indian authors (Springer series).
2005	Successful attempt in establishing Cochrane centre	Second attempt establishing a Cochrane centre/network in India (successful) from Christian Medical College, Vellore, and forming the South Asian Cochrane Network.
2005 to 2020	Cochrane South Asia region: Christian Medical College (CMC), Vellore https://www.cmch-vellore.edu/	Forerunner of evidence production and capacity building in evidence synthesis, in India. It hosted the first Cochrane regional centre in the South Asia region. Cochrane South Asia was formed in 2012 at CMC Vellore.
2007	Access to the Cochrane Library	Indian Council of Medical Research (ICMR) procured a subscription to the Cochrane Library, making it freely accessible to Indian researchers. This resulted in an increase in Indian authors (from 11 in 2005 to 272 in February 2014) publishing protocols and full reviews in the Cochrane Library.
2008	EURECA (evidence that is understandable, relevant, extendible, current and appraised) for EBM	Indian Paediatrics journal established a regular section termed ‘“EURECA”’
2008	International Initiative for Impact Evaluations (3ie) https://www.3ieimpact.org/	3ie is an international organization working in the area of evidence synthesis. The organization is responsible for generation and effective use of high-quality evidence to inform decision-making in LMICs. The organization has offices at Washington and London, and its Indian regional office is situated in the national capital i.e. New Delhi.
2012	Advanced Center for Evidence Based Child Health, PGIMER, under the aegis of ICMR http://acebch.org/about-acebch/	This centre focuses on conducting systematic reviews related to child health, building capacity to conduct systematic reviews and to inform evidence-based policies for child health.
2013	Public Health Evidence South Asia (PHESA) http://www.phesa.manipal.edu/Default.aspx	Currently under Prasanna School of Public Health, Manipal Academy of Higher Education, PHESA is a South-Asian satellite of the Cochrane Public Health Group. The focus of PHESA is on producing evidence for policy in the South Asian region and capacity building in the area of evidence synthesis. PHESA is also supporting the Cochrane India network, as the evidence synthesis activities of the Cochrane Affiliate Centre at Manipal Academy of Higher Education, Manipal.
2013	The George Institute for Global Health (TGI) https://www.georgeinstitute.org/	It is a premier institute with a mission to improve the health of millions of people worldwide. The institute has been working in the field of evidence synthesis that helps in guiding policy decisions. The Indian regional office of the TGI India, was established in 2013, at New Delhi, to cater to the needs of the Indian subcontinent.
2014	India’s Ministry of Health & Family Welfare (MoHFW) established a task force on standard treatment guidelines.	To standardize evidence-based clinical management of diseases in India, MoHFW convened a guideline task force.
2015	National Institute for Health and Care Excellence (NICE) International, UK, provided technical assistance to the government of India	To help develop evidence-based national standard treatment guidelines, NICE International, UK is providing technical assistance to MoHFW.
2017	National Health Policy 2017	The Policy recommends the use of evidence and prioritizing the role of the government in shaping health systems in all its dimensions.
2017	Broadening access to the Cochrane Library	Indian Council of Medical Research (ICMR) procured a subscription to the Cochrane Library making it freely accessible to students, practitioners, researchers and patients.
2018	Establishment of Health Technology Assessment in India (HTAIn) https://htain.icmr.org.in/	Established for evaluation and appropriateness and cost-effectiveness of the available and new health technologies in India. HTAIn is functioning under the Department of Health Research, Ministry of Health and Family Welfare, Government of India.
2019 Campbell South Asia 2019	Campbell Collaboration established regional centre in Delhi https://www.campbellcollaboration.org/	The Campbell Collaboration was founded in 2000, when the social and behavioural scientists and social practitioners came together to form a collaboration. It supports production and use of systematic reviews and other evidence synthesis methods for evidence-based policy and practice. The Campbell South Asia regional centre was established in New Delhi, India, in 2019.
2021	The Cochrane India Network https://india.cochrane.org/	This is a network of nine regional Cochrane affiliate centres that are distributed across the country.

With these structures and commitments in place, the question remains whether India has converted this progress and understanding of evidence synthesis into policy development? Evaluation and review of some of the clinical guidelines in the past suggest that India is in a transition phase of guideline development and the country requires more local capacity in evidence search and synthesis to increase the methodological quality and rigour of the clinical guidelines (Sonawane *et al.*, 2015; Bhaumik *et al.*, 2018).

Beyond clinical guidelines, the use of systematic review evidence has been prioritized for framing policies for one of the important components of public health in India, namely the National Health Programmes (NHPs). However, comprehensive information about this use is not available in one place. Although there is information available on the evaluation of some of the clinical guideline documents, there is a gap regarding information about the use of systematic reviews in other, non-clinical NHPs. In this paper, we have widened our scope beyond clinical guidelines to consider the use of systematic review evidence in the NHPs of India that are mentioned on the MoHFW website. Table 2 provides a list of the major NHPs in India that were considered in this paper [Ministry of Health and Family Welfare (MoHFW), not dated; National Health Portal of India, not dated]. Our analysis focuses on the patterns and trends associated with the use of evidence generated from systematic reviews in the Indian NHP policy or guideline documents, which will be useful in creating and revising the guideline documents in the future.

**Table 2.** National Health Programmes in India

Name of the programme or sub-programmes	Details
A) Reproductive Maternal, Neonatal, Child and Adolescent Health (RMNCH + A)	
Reproductive and Child Health (RCH) Programme	The Reproductive and Child Health (RCH) Programme was launched in 1997 with the second phase of the programme, RCH-II, in 2005.
*Sub-programmes of RMNCH + A*	The RMNCH + A strategy was launched in 2013 to provide an understanding of ‘continuum of care’ to ensure equal focus on various life stages.
*1. Reproductive health* Family planning programmes (contraceptives such as oral contraceptive pills, intra-uterine contraceptive devices and sterilization, as well as abortion care)	The National Programme for Family Planning was launched in India in 1952 and was integrated into the RMNCH + A in 2013. The objectives, strategies and activities of the family planning programme aim at achieving the family welfare goals and objectives [reducing crude birth rate (CBR), total fertility rate (TFR) and growth rate] stated in various policy documents such as the National Population Policy, National Health Policy and National Health Mission.
*2. Maternal health and newborn health* Janani Suraksha Yojana (JSY) Janani Shishu Suraksha Karyakram (JSSK) India Newborn Action Plan (INAP)^a^ Dakshata programme Pradhan Mantri Surakshit Matritva Abhiyan (PMSMA) Midwifery services^a^ LaQshya- Quality Improvement Initiative Programmes on nutrition and calcium supplementation^a^, deworming^a^, syphilis screening^a^ during pregnancy/lactation and gestational diabetes mellitus^a^	Schemes for maternal and newborn health such as JSY (launched in 2005) and JSSK (launched in 2011) are an integral part of the National Rural Health Mission (now called National Health Mission) for maternal and newborn health to provide incentives and quality services to mothers and newborns and enabling institutional delivery. The INAP was launched in 2014 and aims at ending preventable newborn deaths and accelerating cost-effective interventions. The Dakshata programme was launched in 2015 to enable the service providers in providing high-quality services during childbirth in institutions to reduce maternal and newborn mortality in the country. PMSMA was launched in 2016 to improve the quality and coverage of antenatal care (ANC) including diagnostics and counselling services. The midwifery services programme was initiated in the year 2007 as a pilot project for strengthening the training of midwives and nurses who play an integral part in maternal and newborn health. LaQshya Quality Improvement Initiative was launched in 2017 to improve the quality of labour rooms in the country. The programmes for supplementation during pregnancy and other programmes, such as deworming and screening for syphilis, were launched after the year 2014 as a component of improving maternal health under RMNCH + A.
*3. Child health* Rashtriya Bal Swasthya Karyakram (RBSK)	The RBSK was launched in 2013 to screen and manage children from birth to 18 years of age for defects at birth, deficiencies, diseases and developmental delays including disabilities.
*4. Adolescent health* Rashtriya Kishor Swasthya Karyakram (RKSK) Weekly iron and folic acid supplementation (WIFS)	The MoHFW launched RKSK in 2014 for improving sexual and reproductive health, nutrition, injuries and violence, non-communicable diseases, mental health and substance misuse among adolescents—male and female, rural and urban, married and unmarried, in and out-of-school adolescents with special focus on marginalized and underserved groups. The MoHFW launched the WIFS programme in 2012 to meet the challenge of high prevalence and incidence of anaemia amongst adolescent girls and boys by targeting school-going adolescent girls and boys in 6th to 12th class in government/government aided/municipal schools and out-of-school adolescent girls.
B) Communicable diseases	
*National AIDS Control Programme (NACP*)*^a^*	Launched in 1992 and since then it has been the major programme for control and management of HIV/AIDS in India. According to the need and strategies, the programme has been revised as NACP II (1999), NACP III (2007–2012) and NACP IV (2012–2017, extended till 2020). The programme achieved the Millenium Development Goal 2015 target of achieving 50% reduction in new infections and AIDS-related deaths. The strategies of the programme have been revised to achieve more comprehensive and effective coverage of AIDS-related services.
*Integrated Disease Surveillance Programme (IDSP)*	The programme was launched in 2004 for quick detection and response to outbreaks. This programme was initiated by the support of the World Bank fund that continued till 2012.
*National Tuberculosis Elimination Programme (NTEP*)*^a^*	Launched as the National Tuberculosis Programme (NTP) in 1962. After a joint review of the programme in 1992 by the Government of India, WHO and Swedish International Development Agency (SIDA), the programme was later revised. Declaration of TB as a global emergency by the WHO and recommendation of DOTS as a treatment strategy coincided with the revitalization of the NTP as the Revised National Tuberculosis Control Programme (RNTCP) in 1993. In 1997, India launched DOTS as a treatment strategy under the RNTCP and by 2005 the entire country was covered by the programme. The second phase of RNTCP commenced from 2006–2011 and the targets were achieved by 2007–2008. The programme was re-named as the National Tuberculosis Elimination Programme (NTEP) in 2020. This aligns with the objective of eliminating TB from India by the year 2025, documented in the National Strategic Plan for Control and Tuberculosis Elimination (2017–2025).
*National Vector Borne Diseases Control Programme (NVBDCP)* Integrated Vector Management National Malaria Eradication Programme National Filariasis Control Programme National Kala-azar Elimination Programme Dengue and Chikungunya^a^ Acute Encephalitis Syndrome (AES)/Japanese Encephalitis National Filariasis Control Programme National Kala-azar Elimination Programme Dengue and Chikungunya AES/Japanese Encephalitis	NVBDCP is the primary programme for prevention and control of vector borne disease in India. It was launched in 2003–2004 after merging the National Anti-malaria Programme, National Filaria Control Programme and Kala Azar Control Programme. Diseases like Japanese Encephalitis and Dengue have also been included in the programme. The programme includes the National Malaria Eradication Programme, Kala-Azar Elimination Programme, National Filaria Control Programme (1955, extended to rural areas in 1994), Japanese Encephalitis Control Programme, Dengue and Dengue Hemorrhagic Fever.
C) Non-communicable diseases (NCDs), injury and trauma (categorized into NCD I and NCD II)	
*NCD I* National Mental Health Programme (NMHP) National programme for health care of the elderly (NPHCE) National Programme for the Prevention and Control of Deafness (NPPCD)	The NMHP was launched in 1982 and was re-strategized in 2003 to include, (1) modernization of state mental hospitals, (2) up-gradating of psychiatric wings and of medical colleges/general hospitals. The NPCHE was launched in 2010–2011 to address the health problems of older adults. This is a State oriented programme with major focus of providing health care facilities to the senior citizens (>60 years old) at primary, secondary and tertiary levels of health care. The NPPCD was launched in 2007 as a pilot programme in 25 districts of 11 States/Union Territories and was expanded to all the states by 2017.
*NCD II:* National Programme for Prevention and Control of Diabetes (NPCDCS), Cancer, Cardiovascular Diseases and Stroke (NPCDCS)^a^ Pradhan Mantri National Dialysis Programme	The NPCDCS was launched in 2011, after the National Cancer Control Programme was launched with the National Programme for Diabetes, Cardiovascular Diseases and Stroke. Recently, other chronic diseases like chronic respiratory diseases and kidney diseases have been included in this programme. Launched in 2016–2017, the Pradhan Mantri National Dialysis Programme is under the public private partnership at the district hospitals.
*National Programme for Prevention and Management of Trauma and Burns injuries*	Initially the programme was launched with two separate components of trauma care and burn injuries. In 2017, both the components of the National Programme for Prevention and Management of Trauma and Burn Injuries were merged as ‘Territory Care Programmes’.
D) Universal Immunization and Pulse Polio Programme	
*Universal Immunization Programme* ^a^	The Universal Immunization Programme (UIP) programme was launched in 1985, with an objective of reducing mortality and morbidity due to the six vaccine-preventable diseases. Since then, the programme has seen changes according to the need and objectives and was added under the National Rural Health Mission in 2005. The Government of India declared 2012 as the ‘year of intensification of routine immunization’, and a commitment towards measles elimination by 2020, under the UIP.
*Pulse Polio Programme*	The programme was started in 2005, as a specific programme for polio control and elimination in addition to the UIP. Due to continued efforts and proper implementation, India reported the last case of polio in 2011 and was removed from the list of ‘endemic to polio’ countries in 2012. India was certified as wild poliovirus free in 2014.
E) Other National Health Programmes	
*National Programme for Prevention and Control of Fluorosis (NPPCF)* *National Tobacco Control Programme*	The NPPCF programme was launched in 2008–2009 and since then has been expanding in a phased manner. NPPCF addresses the problem of fluorosis due to high fluoride intake and works via the strategies of surveillance, capacity building, diagnosis, health education and management. For the prevention and control of tobacco use.

a Programmes that used systematic review evidence.

Source: The Ministry of Health and Family Welfare and National Health Mission (https://main.mohfw.gov.in/; https://nhm.gov.in).

## Methods

A study design of stand-alone document analysis was applied to answer the question of whether India’s NHP guideline documents are explicitly informed by evidence syntheses. The methods followed recommendations from a qualitative systematic review of document analysis in health policy analysis studies in low- and middle-income countries (LMICs) (Kayesa and Shung-King, 2021) adopting clear inclusion criteria for documents, and clear procedures for identifying documents, coding them and extracting data; applying a clear analytical framework to analyse the role of systematic reviews cited in policy documents; and presenting the findings of each stage of the process from searching for documents to answering the research question.

A targeted search was conducted to identify the documents providing information about the use of systematic reviews in NHPs of India. Documents sought for analysis were the guideline documents produced by the NHPs in India (listed in Table 2) that cited systematic reviews. These were identified by searching official information sources or websites of the NHPs. Official websites of NHPs were searched for guidelines or policies that were based on systematic reviews i.e. the guideline documents that had cited systematic reviews with proper bibliographic information. The above-mentioned websites were accessed via the official website for the MoHFW, Government of India, as all the NHPs are linked to this ministry website. As the MoHFW is a central nodal ministry for health-related information or data in India, the NHPs mentioned on this website were considered in this document analysis. Searches were conducted in December 2020 and January 2021.

**Figure 1.**
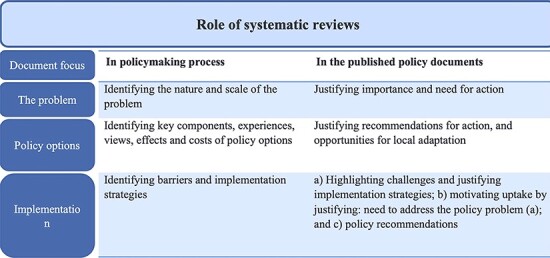
Framework to classify systematic review evidence use in policy documents (adapted from Lavis, 2009)

Documents available in the official government websites were screened at the time of searching, and information about any systematic review evidence used in framing of guidelines and policies, for that NHP, was extracted. We classified the systematic review evidence according to its role in the policymaking process. This was done by adapting the framework given by Lavis (2009) on the role and use of systematic reviews in the policymaking process. We used Lavis’ framework and adapted it for classifying systematic reviews according to their role in the published policy documents (Figure 1). Specifically, we sought systematic reviews that informed three steps in developing policy. The first step is fulfilled by systematic reviews that provided information about the nature and scale of a policy problem and thereby justify its importance and the need for policy attention. These may be systematic reviews of observational or qualitative studies. The second step calls for systematic reviews that assess policy options in terms of effectiveness (from controlled trials), harm (from observational studies), cost-effectiveness (economic evaluations), and how or why interventions work, or not (often from mixed methods studies). The last step is implementation, which draws on systematic reviews of implementation barriers (from observational or qualitative studies) or effective solutions (from controlled trials).

More information is given in the Results section. This is a document analysis of NHP guideline documents, we did not perform a systematic review as our objective was not to synthesize data available from these documents, but to summarize the information available on use of systematic review evidence available from the NHP guideline documents.

## Results

### Use of evidence to inform policy decisions in NHPs of India

Over the years, the NHPs of India have used a range of systematic review evidence to inform the various steps in framing guidelines and policies. In the section below, we have classified the identified systematic review evidence cited in the guideline documents of the NHPs as: (1) systematic reviews justifying the importance and need for action; (2) systematic reviews justifying recommendations for action, and opportunities for local adaptation; and (3) systematic reviews informing implementation. Detailed information on the identified evidence is given in Table 3.

**Table 3.** Classification of the identified systematic review evidence according to its use in the policy process (ordered by stage of policy development first, followed by year)

Document focus	National Health Programme	Year	Policy document partners/contributors/ supporters	Systematic reviews cited
1. The problem	Reproductive-Maternal- Neonatal-Child and Adolescent Health (RMNCH + A) strategy components	2014	National guidelines on deworming in pregnancy. (Ministry of Health and Family Welfare, 2014a) *Contributors: Other Indian clinicians and researchers; Jhpiego; UNICEF*	These guidelines cite a systematic review that quantifies the association between hookworm infection and anaemia (Brooker *et al.*, 2008) *Data sources: India + international; Author location^b^: International*
1. The problem	RMNCH + A strategy components	2014	India Newborn Action Plan (Ministry of Health and Family Welfare, 2014b) *Technical support from UNICEF, Save the Children, WHO, USAID, BMGF, DFID and NIPI*	These guidelines cited systematic reviews characterizing the health burden of conditions related to childbirth, particularly stillbirth (Cousens et al., 2011)^a^, child mortality (Liu *et al.,* 2012)^a^, small for gestational age (Lee *et al.*, 2013)^a^, neurodevelopmental impairment (Blencowe *et al.*, 2013) and the timing of overall and cause-specific neonatal deaths (Sankar *et al.*, 2016) *Some citations were directly linked to statements in the main text* *Data sources: India and international; Author location: Indian and international*
1. The problem	National Tuberculosis Elimination Programme (NTEP)	2017	Final Report Joint Assessment of the Tuberculosis Diagnostic Network of India (Ministry of Health and Family Welfare, 2017b) *Contributors: India’s Central TB Division (Directorate General of Health Services, Ministry of Health and Family Welfare), USAID, BMGF, PATH, FIND, KHPT, KNCV Tuberculosis Foundation, CDC, REACH, WHO, clinicians and staff at health facilities*	A systematic review (Subbaraman *et al.*, 2016) was used by the tuberculosis (TB) diagnostic network of India to explain and quantify the problem of attrition in the TB diagnostic cascade in the country. *Data sources: India* *Author location: Indian and international*
1. The problem	National Programme for Prevention and Control of Diabetes, Cancer, Cardiovascular Diseases and Stroke (NPCDCS)	2017	National framework for joint TB–diabetes collaborative activities: Revised National Tuberculosis Control Programme (RNTCP) (Ministry of Health and Family Welfare, 2017a) *Supported by: National Institute of Tuberculosis and Respiratory Diseases, ICMR, The UNION and World Health Organization Country Office*	The document cites two systematic reviews about interactions between diabetes and TB (Jeon and Murray, 2008; Baker *et al.*, 2011) that justify the need for policy action. *Citations were directly linked to statements in the main text* *Data sources: India + international* *Author location: International*
1. The problem	National Vector Borne Disease Control Programme (NVBDCP)	2020	National guidelines for dengue case management during Covid-19 pandemic (Ministry of Health and Family Welfare, 2020a) *Experts: AIIMS; Maulana Azad Medical College; Vardhman Mahavir Medical College; LHMC*	Evidence in the form of a systematic review (Lansbury *et al.*, 2020) has contributed to the national guidelines for dengue case management during Covid-19 pandemic. *Data sources: International* *Author location: International*
2. Policy and programme options	RMNCH + A strategy components	2014	National guidelines on deworming in pregnancy (Ministry of Health and Family Welfare, 2014a) *Contributors: AIIMS; many Indian clinicians and scientists; Jhpiego; UNICEF*	These guidelines cite a systematic review on the efficacy of albendazole, mebendazole and pyrantel pamoate against soil-transmitted helminth infections (Keiser and Utzinger, 2008). *Data sources: International* *Author location: International*
2. Policy and programme options	RMNCH + A strategy components	2014	India Newborn Action Plan (Ministry of Health and Family Welfare, 2014b) *Technical support from UNICEF, Save the Children, WHO, USAID, BMGF, DFID and NIPI*	These guidelines cited a review of systematic reviews on effectiveness and cost-effectiveness of preconception, antenatal, intrapartum, and postnatal interventions for reduction in stillbirths ([Bibr R9][Bibr R9]). *Data sources: India + international* *Author location: India + international*
2. Policy and programme options	RMNCH + A strategy components	2014	Screening for syphilis in pregnancy (Ministry of Health and Family Welfare, 2014c) *Supported by: National AIDS Control Organization, institutional experts and development partners*	These guidelines referenced a systematic review assessing drug safety (Galvao *et al.*, 2013) *Citation was directly linked to the statement in the main text* *Data sources: India + international* *Author location: International*
2. Policy and programme options	RMNCH + A strategy components	2014	The national guidelines for calcium supplementation during pregnancy and lactation (Ministry of Health and Family Welfare, 2014d) *Contributors: Indian clinicians and scientists; AIIMS; Jhpiego; UNICEF*	The guidelines have been prepared based on the recommendations of a national expert group and available national/international evidence consisting of WHO guideline and a Cochrane review (Hofmeyr *et al.*, 2018) about the effects of calcium supplementation. *Citations were directly linked to the statement in the main text* *Data sources: India + international* *Author location: International*
2. Policy and programme options	RMNCH + A strategy components	2014	The operational guidelines on Kangaroo Mother Care (KMC) and optimal feeding of low-birth-weight infants (Ministry of Health and Family Welfare, 2014e) *Contributors: Indian clinicians and scientists; USAID-MCHIP*	The guidelines utilized a Cochrane review (Conde Agudelo *et al.*, 2012) concluding that KMC was effective and safe for clinically stable preterm newborns. *Citation was directly linked to the statement in the main text* *Data sources: India + international* *Author location: International*
2. Policy and programme options	RMNCH + A strategy components	2014	Operational guidelines on Injection Vitamin K Prophylaxis at Birth (in facilities) (Ministry of Health and Family Welfare, 2014f) *Experts: AIIMS, UNICEF, NIPI, KSCH*	The guideline suggested that a Cochrane review from 2000–2003 supports the use of vitamin K for all newborns; however, the reference to the Cochrane review is not listed in the bibliography of the guideline. *No information on data sources or author location due to absence of systematic review references in the document*
2. Policy and programme options	RMNCH + A strategy components	2014	‘Operational Guidelines: Use of Antenatal Corticosteroids in Preterm Labour’ (Ministry of Health and Family Welfare, 2014g) *Technical experts: AIIMS; MAMC; KSCH; MGIMS; LHMC; UNICEF; USAID–MCHIP; Jamia Nursing College.*	The guideline states that ‘a single course of corticosteroid therapy for preterm birth does not appear to be associated with any significant short-term maternal or foetal adverse effects’, but the systematic review is not listed in the bibliography of the guideline. *No information on data sources or author location due to absence of systematic review references in the document*
2. Policy and programme options	RMNCH + A strategy components	2017	National Guidelines on Lactation Management Centres in Public Health Facilities (Ministry of Health and Family Welfare, 2017c) *Contributors: AIIMS; MAMC; LHMC; NIPI; UNICEF*	These guidelines cited four systematic reviews (McGuire and Anthony, 2003; Boyd *et al.*, 2007; Quigley *et al.*, 2007; [Bibr R3][Bibr R3]) supporting protection offered by human milk for preterm infants against necrotizing enterocolitis compared to formula milk. *Citation was directly linked to the statement in the main text* *Data sources: International; Author location: International*
2. Policy and programme options	National AIDS Control Programme (NACP)	2016	Prevention and Management of tuberculosis in people living with HIV at Anti-retroviral therapy Centres (Ministry of Health and Family Welfare, 2016a) *NACO and the CTD prepared the guidelines with technical support from The CDC—CDC-DGHT India, SHARE India, and WHO India.*	This guideline states the importance of intensified case finding of TB at anti-retroviral therapy centres based on four symptoms (also known as the 4S complex) i.e. current cough of any duration, fever of any duration, weight loss or night sweats. This guideline is strengthened by the evidence from a meta-analysis (Getahun *et al.*, 2011) reporting that the sensitivity of the 4S complex was 85%. *Citation was directly linked to the statement in the main text* *Data sources: International; Author location: International*
2. Policy and programme options	NTEP	2016	INDEX-TB Guidelines: guidelines of extra-pulmonary tuberculosis in India (Ministry of Health and Family Welfare, 2016b) *Partners: Global Health Advocates, India; Cochrane Infectious Diseases Group; Cochrane South Asia; WHO country office for India*	The use of steroids in HIV-negative patients with TB pericarditis with pericardial effusion is recommended in this guideline. This recommendation was based on the evidence from a Cochrane systematic review (Wiysonge *et al.*, 2017) that reported a decrease in mortality due to pericarditis among HIV-negative patients. Evidence from the same review was used to recommend use of corticosteroids in HIV-positive patients with pericarditis. *Data sources: International; Author location: International* Evidence from a Cochrane systematic review (Prasad *et al.*, 2016) was used for recommending the use of corticosteroids for treatment of tubercular meningitis in HIV-negative people, with a minimum duration of 4 weeks. The same review also supported recommendations suggesting the use of corticosteroids, for treating tubercular meningitis in HIV-positive people where other life-threatening opportunistic infections are absent. This evidence was used as a recommendation in the INDEX-TB guidelines. *Data sources: India + international; Author location: India + international* A systematic review was performed for one of the important recommendations in the guidelines about the duration of treatment in case of tubercular meningitis. The review was conducted using Cochrane methodology under the guidance of the methodology group, leading to the recommendation that tubercular meningitis should be treated with first line standard anti-tubercular treatment for at least 9 months. *Citations were directly linked to the statement in the main text*
2. Policy and programme options	NTEP	2016	INDEX-TB Guidelines: guidelines of extra-pulmonary tuberculosis in India (Ministry of Health and Family Welfare, 2016b) *Partners: Global Health Advocates, India; Cochrane Infectious Diseases Group; Cochrane South Asia; WHO country office for India*	These guidelines were based on evidence from already existing or new systematic reviews conducted by the guideline development team. Cochrane methodology was used for conducting the systematic reviews and the Cochrane South Asia network and Cochrane Infectious Disease group provided technical and methodological expertise for the same. The guideline group considered evidence from a systematic review (Denkinger *et al.*, 2014) on the specificity and sensitivity of XPERT MTB/RIF (a diagnostic test for extra-pulmonary TB), to recommend use of the same for EPTB cases. *Citations were directly linked to the statement in the main text* *Data sources: India + international; Author location: International*
2. Policy and programme options	NTEP	2021	Programmatic Management of Drug Resistant Tuberculosis in India (Ministry of Health and Family Welfare, 2021) *Developed by: Government of India (GoI), WHO India and key technical and development partners*	The guideline mentions that closed contacts of the index TB patient should be identified via rigorous contact tracing, to control TB transmission. This strategy was based on evidence from a systematic review and meta-analysis (Morrison *et al.*, 2008) that had provided information on the yield of TB among contacts and concluded that contact investigation is an important tool for improving early case detection and reducing transmission of TB in high-burden areas or countries. *Data sources: India + international; Author location: International*
2. Policy and programme options	Universal Immunization Programme (UIP)	2018	Guidelines on use of syrup paracetamol following vaccinations (Ministry of Health and Family Welfare, not dated) *Contributors: Indian clinicians and scientists*	*This guideline mentioned two systematic reviews reporting the benefits of prophylactic and therapeutic use of paracetamol for relief in local and systemic symptoms after primary vaccinations and reduction in the antibody responses to some vaccine antigens. However, this guideline did not provide the references of these two systematic reviews. No information on data sources or author location due to absence of systematic review references in the document. No information on data sources or author location due to absence of systematic review references in the document*
2. Policy and programme options	NACP	2018	National Technical Guidelines on Anti-retroviral Treatment (Ministry of Health and Family Welfare, 2018a) *Contributors: Clinicians and scientists from India and USA*	The rationale for using a lopinair-based regimen for children <3 years of age was supported by the two systematic reviews (references for these systematic reviews were not given in the bibliography of the report, therefore not cited here). These systematic reviews reported that a lopinair-based regimen was effective, and it had a low failure rate. *No information on data sources or author location due to absence of systematic review references in the document*
2. Policy and programme options	RMNCH + A strategy components	2018	The technical and operational guidelines for diagnosis and management of gestational diabetes mellitus (GDM) (Ministry of Health and Family Welfare, 2018b) *Contributors: MoHFW, UNICEF, JHPIEGO, IPE Global, AIIMS, International Diabetes Federation, FOGSI and others*	This guideline cited a systematic review (Farrar *et al.*, 2011) that supported the two-step screening approach for pregnant women at 11 to 14 weeks’ gestation who were at a lower risk of being diagnosed with GDM as compared to the one-step approach. The safety and effectiveness of oral hypoglycaemic drugs recommending use of metformin for GDM management after 20 weeks of gestation in the guideline was based on a systematic review (Gui *et al.*, 2013). *Data sources: International; Author location: International*
2. Policy and programme options	RMNCH + A strategy components	2018	Guidelines for midwifery services in India, 2018 (Ministry of Health and Family Welfare, 2018c) *Contributors: Indian clinicians and scientists; BMGF; JHPIEGO; IPE Global; White Ribbon Alliance for Safe Motherhood India, Laerdal Global Health; UNFPA; UNICEF; WHO*	This guideline cited a Cochrane review that provided evidence on the midwife-led continuity of care for reduction in episiotomy, instrumental birth or use of pain relief and improved psychological support for women (Sandall *et al.*, 2016). *Data sources: International; Author location: International*
2. Policy and programme options	NTEP	2020	The India TB report 2020 (Ministry of Health and Family Welfare, 2020b) *Contributors: Central TB division, Ministry of Health and Family Welfare*	This report describes research having an impact on various RNTCP strategies or policies, e.g. diagnostic and treatment. This report highlights the role of systematic review evidence in framing public health policies and provides information about a systematic review that had an impact on framing of the TB treatment policy. This systematic review contributed to changing the TB treatment regimen from an intermittent to a daily regimen, i.e. DOTS. The systematic review (Azhar, 2012) concluded that there was limited evidence comparing a DOTS category 2 regimen with self-administered treatment in re-treatment cases. *Data sources: India; Author location: India*
3. Implementation	RMNCH + A strategy components	2018	Guidelines for midwifery services in India, 2018 (Ministry of Health and Family Welfare, 2018c) *Contributors: Indian clinicians and scientists; BMGF; JHPIEGO; IPE Global; White Ribbon Alliance for Safe Motherhood India, Laerdal Global Health; UNFPA; UNICEF; WHO*	The guidelines are based on a rapid evidence review of the one-year *Nurse Practitioner in Midwifery curriculum* and the *International Confederation of Midwives competencies*, and a National Midwifery Task Force consultation. The exercise concluded that additional post-basic education and training is required in order to build competencies of midwives to deliver quality care. The guidelines included an evidence-supported 18-month training (a 6-month training, in addition to the earlier 1-year training) encompassing both theory and practical sessions, as well as competency-based sessions for providing training to the midwives. *Data sources: India; Author location: India*
3. Implementation	NACP	2007	The NACP training of medical officers on HIV care and treatment (including Antiretroviral Therapy (ART)) (Ministry of Health and Family Welfare, 2007) *Partners: WHO; CDC* *Contributors: Indian clinicians*	This training manual has used evidence from systematic reviews to provide information about the efficacy and safety of TB prophylactic drugs, (Wilkinson *et al.*, 1998) and the interactions between classical sexually transmitted diseases and HIV (Røttingen *et al.*, 2001) *Citations were directly linked to statements in the main text* *Data sources: India + international; Author location: International*
3. Implementation	NPCDCS	2016	Reducing risk factors for Non-Communicable Diseases (NCDs) in Primary Care: Training manual for Medical Officers (Ministry of Health and Family Welfare, 2016c) *Contributors: Indian and international clinicians and scientists; WHO*	The document cited three systematic reviews: two defined the problem (Roest *et al.*, 2010; DiMatteo *et al.*, 2000) and the third assessed policy options (Lachat *et al.*, 2013). There were no clear statements on the use of systematic review evidence for the development of the training manual. *Data sources: India + international; Author location: International*
3. Implementation	UIP	2019	Operational Guidelines— Introduction of Rotavirus Vaccine in the Universal Immunization Programme (Ministry of Health and Family Welfare, 2019) *Contributions from: JSI, WHO, UNICEF, UNDP, ITSU, NCCVMRC and others*	To plan for expansion of rotavirus vaccines to other states of India, the mission steering committee of NHM, ICMR carried out a programme implementation review (a rapid assessment of the UIP conducted in the four phase-1 states of India) to generate evidence for the MoHFW, Government of India. The report cited systematic reviews about the nature and scale of the problem (Tate *et al.*, 2009; Liu *et al.*, 2012). *Data sources: India + international; Author location: International*

a Review of systematic reviews and systematic analysis (involving systematic methods for literature search/ reviews and/or policy analysis).

b Author location is based on the institutional affiliations of the systematic review authors.

NACP: National AIDS Control Programme; NPCDCS: National Programme for Prevention and Control of Diabetes, Cancer, Cardiovascular Diseases and Stroke; NTEP: National Tuberculosis Elimination Programme; RMNCAH + A: Reproductive-Maternal- Neonatal-Child and Adolescent Health; UIP: Universal Immunization Programme. AIIMS: All India Institute of Medical Sciences; BMGF: Bill and Melinda Gates Foundation; CDC: Centre for Disease Control and Prevention; CDC-DGHT: CDC-Division of Global Health and tuberculosis; CTD: Central TB Division; DFID: Department for International Development; DOTS: Directly Observed Treatment, Short-course; EPTB: Extrapulmonary tuberculosis; FIND: Foundation for Innovative New Diagnostics; FOGSI: Federation of Obstetric and Gynaecological Societies of India; GDM: gestational diabetes mellitus; ICMR: Indian Council of Medical Research; Jhpiego: Johns Hopkins Program for International Education in Gynaecology and Obstetrics; KHPT: Karnataka Health Promotion Trust; KMC: Kangaroo Mother Care; KSCH: Kalawati Saran Children’s Hospital; LHMC: Lady Hardinge Medical College; MAMC: Maulana Azad Medical College; MoHFF: Ministry of Health and Family Welfare; NACO: National AIDS Control Organization; NHM: National Health Mission; NIPI: Norway India partnership Initiative; REACH: Resource Group for Education and Advocacy for Community Health; SHARE: Society for Health Allied Research Education; TB: Tuberculosis; UNFPA: United Nations Population Fund; UNICEF: United Nations Children’s Fund; USAID: US Agency for International Development; WHO: World Health Organization.

#### Systematic review evidence for justifying the importance and need for action

Four NHPs, namely Reproductive, Maternal, Neonatal-Child and Adolescent Health (RMNCH + A), National Tuberculosis Elimination Programme (NTEP), Non-Communicable Diseases, Injury & Trauma and National Vector Borne Disease Control Programme (NVBDCP), mentioned or cited the use of systematic review evidence in their guideline documents for justifying the importance and need for action. Five published guideline documents from the aforementioned four NHPs, namely ‘*National Guidelines for Deworming in Pregnancy*’ (2014), ‘*India Newborn Action Plan*’ (2014), ‘*Final Report joint assessment of the tuberculosis diagnostic network of India*’ (2017), ‘*National framework for joint TB diabetes collaborative activities-RNTCP*’ (2017) and ‘*National guidelines for dengue case management during Covid-19 Pandemic*’ (2020), justified the importance and need for action via systematic review evidence that provided information on the nature and scale of the problem (Ministry of Health and Family Welfare, 2014a,b; 2017a,b; 2020a).

#### Systematic review evidence for justifying recommendations for action, and opportunities for local adaptation

Sixteen published guideline documents from four NHPs [RMNCH + A, Universal Immunization Programme (UIP), NTEP and National AIDS Control Programme (NACP)] used systematic review evidence for justifying recommendations for action and opportunities for local adaptation. These 16 published guideline documents cited systematic reviews that provided information on the different programme and policy options applicable for the specific NHPs (Ministry of Health and Family Welfare, 2014a,b,c,d,e,f,g; 2016a,b; 2017c; 2018a,b,c; 2020b; 2021; not dated). More information on the guideline document and the systematic review evidence used is given in Table 3.

#### Systematic review evidence used to inform implementation

Four published guideline documents from RMNCH + A, NACP, National Programme for Prevention and Control of Cancer, Diabetes, Cardiovascular diseases and Stroke (NPCDCS), and UIP used systematic review evidence for informing implementation. In line with Lavis’ framework for evidence use in policy (Lavis, 2009), the ‘*Operational Guidelines—Introduction of Rotavirus Vaccine in the Universal Immunization Programme*’ (2019) justifies the implementation strategies for the process of scaling-up of the rotavirus vaccine, therefore, supporting implementation (Ministry of Health and Family Welfare, 2019). The ‘*Guidelines for midwifery services in India*’ (2018) (Ministry of Health and Family Welfare, 2018c), evidenced the challenges of implementation in a slightly different way, with a rapid evidence review of the 1-year Nurse Practitioner in Midwifery curriculum and the International Confederation of Midwives competencies, and a National Midwifery Task Force consultation. The exercise concluded that additional post-basic education and training is required in order to build competencies of midwives to deliver quality care. Finally, two other implementation documents, the ‘*NACP training of medical officers on HIV care and treatment (including ART)*’ (2007), and ‘*Reducing risk factors for Non-Communicable Diseases (NCDs) in Primary Care: Training manual for Medical Officers*’ (2016), cited systematic reviews that provided information on the extent of the problem and assessed different policy options (Ministry of Health and Family Welfare, 2007; 2016c). As these guideline documents were used as training manuals for the medical officers, this evidence may have been cited to justify the guidance and motivate uptake.

### How the use of systematic review evidence varied across national health programmes of India

The section above classifies systematic review evidence according to its role in the published NHP guideline documents. In this section, we have elaborated more on the use of systematic review evidence by the NHPs of India. We found only six NHPs providing information about the use of systematic reviews in the guidelines (see Table 3). Additionally, not all the sub-themes or programmes, within these six programmes, provided information on systematic review use (see Table 2).

Systematic review evidence was mostly visible in guidelines or policies addressing maternal and newborn health (RMNCH + A), communicable diseases and immunization. Most systematic reviews cited in the guideline documents provided information on assessing policy options and were used for justifying recommendations for actions and opportunities for local adaptation. Guideline or policy documents addressing implementation cited systematic reviews of epidemiology about the problems and policy options more often than systematic reviews about implementation.

Only 9 of the 22 guideline documents (41%) consistently linked systematic reviews to the relevant statements in the document (Table 3). Other times they appear in a list of additional readings towards the end of the document.

Indigenization and local adaptation of the systematic review evidence is a vital component in policymaking. We found that for clinical programmes and policies, global systematic review evidence was used and cited in the Indian guidelines and many of these included Indian data. However, for some of the non-clinical guidelines, where the context may be particularly influential in how interventions work, the lack of Indian data is a concern. The INDEX-TB guidelines (Ministry of Health and Family Welfare, 2016b) considered the importance of using local evidence for guideline development. Indeed, the guideline development team acknowledged the absence of context-specific evidence and therefore downgraded the global evidence if it did not include Indian data. Generally, Indian data were mostly used in systematic reviews defining the extent of the policy problem rather than for assessing the different policy options.

Additionally, it was observed that none of the NHP guideline documents drew on synthesis of qualitative research that could justify recommendations for action and identify opportunities for local adaptation or inform implementation.

Important global events and international funding paved the way for the creation of some of the NHPs and their use of systematic review evidence. The NACP was exceptionally early in citing systematic reviews in guideline documents. NACP I & II were initiated after World Bank funding in 1992 and 1999, long before systematic reviews were commonly used for policy (Ministry of Health and Family Welfare, 2010). Another early example was the ‘Revised National Tuberculosis Control Programme’ (National Health Portal of India, not dated), triggered by the declaration of tuberculosis (TB) as a global emergency, which included a training manual prepared in partnership with WHO and Centres for Disease Control in 2007 (Ministry of Health and Family Welfare, 2007) that cited systematic review evidence by Wilkinson *et al.* (1998) and Røttingen *et al.* (2001) about the efficacy and safety of TB prophylactic drugs and the interactions between classical sexually transmitted diseases and HIV.

**Figure 2.**
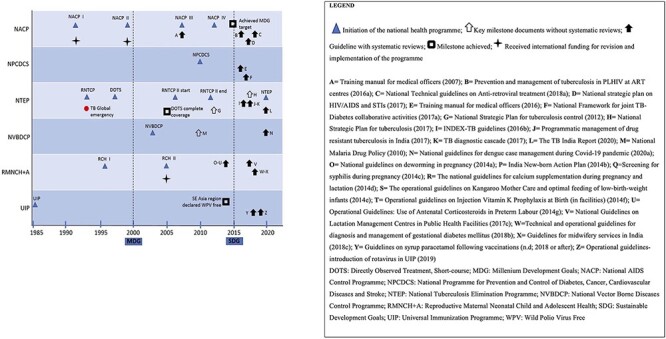
Use of systematic reviews by different National Health Programmes. PLHIV, People Living with HIV; STI, Sexually Transmitted Infection; n.d., not dated; MDG, Millenium Development Goals; SDG, Sustainable Development Goals.

In contrast, most of the guideline documents citing systematic reviews were published after 2014, with major clusters in 2016 and 2017 (see Figure 2). For instance, the Reproductive and Child Health (RCH) I programme was launched in 1996 and revised as RCH II, at the time when the Bill and Melinda Gates Foundation funding for maternal and child health was announced in 2005 (PATH, 2005). However, guideline documents in this programme citing systematic reviews appeared as a cluster in 2014, with a higher frequency in 2017–2018. Two other programmes, NACP and NTEP [previously Revised National Tuberculosis Control Programme (RNTCP)], similarly demonstrated more recent use of systematic review evidence in most of their guideline documents when in 2017 they released their national strategic plans, respectively for AIDS control and TB elimination by 2025. More information about the important milestones and factors related to time that might have influenced the use of systematic reviews by the guideline documents is presented in Figure 2.

The growing visibility of systematic reviews in Indian guideline documents since 2014 coincided with the growth of evidence-informed medicine and evidence synthesis centres in the country (Table 1) and reflects the global trends for producing systematic reviews of health research, first addressing clinical practice in the area of perinatal care and epidemiology (cited in Indian policy documents from 2014), and subsequently health systems research such as continuity of care and screening programmes (cited in Indian policy documents from 2017).

## Discussion

### Summary of findings

This paper focuses on the use of systematic review evidence in formulating public health policies for NHPs in India and the overall evolution of the evidence policy system in India. Our findings show that over the years, India has consistently invested in developing new centres for producing systematic reviews and capacity building in evidence synthesis. Additionally, since 2014, the visibility of evidence has increased in the Indian guideline documents, specifically for six NHPs i.e. NTEP, NACP, NVBDCP, NPCDCS, UIP and RMNCH + A. Overall, citing systematic reviews in guideline or policy documents appears to be a recent development, possibly influenced by international sources of funding. However, proper referencing of the systematic reviews in the guideline documents remains an area of concern because only 9 documents out of 22 guideline documents, identified from the above six NHPs, provided systematic review citations linked to statements in the text. Generally, the systematic review evidence cited in the guideline documents was international, and many of these systematic reviews included Indian data. Indian data were used by more systematic reviews providing information about the problem than those assessing policy options. The lack of Indian data in some systematic reviews providing information on non-clinical, context specific research questions is a concern.

### Comparison with the wider literature

Our findings show India’s evolving system for evidence-informed policy. This evolution can be attributed to the country’s long-term investment in two key complementary components of the evidence system: (1) systematic review generation centres; and (2) commitment to evidence-informed policy development. Theoretical literature offers different models for how these key components work together to support KTA (Best and Holmes, 2010). The knowledge-push model of knowledge translation describes knowledge generated by researchers being packaged as systematic reviews, policy briefs, documentaries etc. for different stakeholders. India’s growing commitment to this model is visible in the increasing number of centres committed to production and dissemination of systematic reviews (Table 1). The push model was further strengthened by formation of the Cochrane India Network in 2021, which includes in its objectives the synthesis of evidence for India and South Asia and dissemination of this evidence in local languages. However, there are still challenges encountered when using the global stock of systematic reviews because they contain limited data from India, so methods are needed to translate this global evidence for use in India.

The demand-pull model of knowledge translation focuses on research users demanding ‘evidence’ for specific policy questions from the researchers, so that the policy decision is evidence informed. Supporting this principle of the demand-pull model of KTA is the growing commitment in India to developing evidence-informed guidance, expressed by guideline groups framing their questions and drawing on existing systematic reviews or commissioning new systematic reviews [e.g. INDEX-TB Guidelines (Ministry of Health and Family Welfare, 2016b)] or tailoring guidance from elsewhere for India (Kattumuri, 2015). The formation of the Health Technology Assessment in India (HTAIn) board strengthened the demand for evidence by the research users. Selected research institutions and researchers in the country produce evidence for the HTAIn board for specific policy questions framed by the HTAIn board (on demand of the decision makers) (Table 1).

The relational model of KTA focuses on linkage exchange, collaboration and shared learning among different stakeholders. This model is apparent where policy makers, clinicians and researchers work together to develop policy documents (guideline documents mentioned in Table 3) and include policy group members from Cochrane entities (Ministry of Health and Family Welfare, 2016b). Informal exchange, a component of the relational model, is apparent from two examples of a systematic review being cited in a policy document before publication: Sankar *et al.* (2016) cited in India Newborn Action Plan (Ministry of Health and Family Welfare, 2014b); and a Cochrane review, Wiysonge *et al.* (2017) cited in INDEX-TB guidelines (Ministry of Health and Family Welfare, 2016b)) (Table 3). Systematic reviews in progress were readily accessible when authors (Ministry of Health and Family Welfare, 2014b) or Cochrane editors (Ministry of Health and Family Welfare, 2016b) were members of the guideline group. The relational model is a good choice for knowledge translation when local or contextual knowledge is considered for adapting evidence-informed decisions. This was visible in the INDEX-TB guidelines (Ministry of Health and Family Welfare, 2016b). Evidence from global reviews were studied and downgraded by the guideline development team before framing policy documents for India.

Systems thinking models for KTA originally focused on how to solve policy problems with all the key stakeholders participating as active collaborators and their organizations investing time and resources in supporting organizational change (Best and Holmes, 2010). Systems thinking has also been applied to building an evidence and policy system itself, with evidence supporting three important components for strengthening use of evidence (Langer *et al.*, 2016). The first essential component for use of research evidence is access to that evidence. India made an important step forward in 2017 when a new licensing agreement gave its students, practitioners, researchers and patients access to the systematic reviews for research addressing healthcare interventions through the Cochrane Library (Cochrane, 2017). The second essential component is decision-makers with skills to access and make sense of evidence (such as critical appraisal training programmes). India began such training programmes for clinicians with INCLEN in 1993 (Table 1). More recently, training in evidence use has reached hundreds of civil servants in India (Harvard Kennedy School, 2018). The third essential component is fostering changes to decision-making structures and processes. This is seen in India with the growth of systematic review centres from 2005 onwards and, in 2013, the MoHFW establishing a guideline task force to standardize evidence-based clinical management of diseases (Table 1). Development of the National Health Policy in 2017 (by the government of India) that prioritizes evidence use adds to this component. Sriram *et al.* (2018) described the enabling contextual factors and supporting interests, and the challenges in the form of complex evidence-to-policy processes and institutional arrangements that would allow both legitimacy and independence.

Global literature also provides information on the current state and evolution of evidence policy systems in other LMICs such as Uganda and Cameroon, where the climate for evidence-informed health policy system has improved over the years. As in India, this change can also be attributed to involvement of external agencies or donors, and over the years the policy documents of these two countries have also increased the usage of scientific evidence (Ongolo-Zogo *et al.*, 2015). A similar study to evaluate the use of evidence in MNCH policy documents was conducted in Nigeria, which reported that the policy documents were prepared in consultation with various stakeholders and external partners. Also, the visibility of evidence in the documents increased post-2015 (Uneke *et al.*, 2017). Studies from other LMICs such as Cambodia, Iran and Pakistan (with contexts similar to India), while explaining the environment, barriers and mechanism of evidence-informed health policymaking in these countries, recommend the increased evidence generation and building capacities of different stakeholders including policymakers to understand the use of evidence in policy (Haq *et al.*, 2017; Liverani *et al.*, 2018; Majdzadeh *et al.*, 2022).

Greater visibility of Indian data in systematic reviews characterizing policy problems rather than considering policy options may indicate a lack of high-quality primary research assessing the effects of policy options in India (Goenka *et al.*, 2019), or poor visibility in academic journals of research from India (SinGH, 2020) and other LMICs (Plancikova *et al.*, 2021).

Additionally, our specific findings on use of systematic review evidence by the NHP guidelines concur with other global literature providing information on the use of systematic reviews in the guideline documents. As it was seen in our analysis that only 9 guideline documents (41%) consistently linked systematic reviews to the relevant statements in the document, similar results have been reported by related global literature. Limited or unsystematic focus on systematic reviews for guideline development is not unusual. A global survey found that few guidelines published in 2017 and 2018 based their recommendations on systematic reviews (32%) or systematic overviews (2%); notable exceptions were those prepared by the WHO and two high-income guideline producers (Lunny *et al.*, 2021). Similar comparisons can be drawn on the use of qualitative synthesis by the guideline documents. While our findings reported that none of the Indian NHP guidelines included qualitative evidence, global literature indicates the use of qualitative synthesis by only 22% of guidelines (Lunny *et al.*, 2021). This could be due to the low recognition and value attached to qualitative research in health systems research (Daniels *et al.*, 2016) and underrepresentation of qualitative research in medical journals (Shuval *et al.*, 2011). Lack of qualitative research in the guideline documents may also indicate a lack of awareness of the availability and value of qualitative synthesis, a methodology that has developed more recently than systematic reviews of the effects of policy options.

### Strengths and limitations

To the best of our knowledge, this is a novel document analysis that comprehensively searched all official websites for the guidelines, and documents, to identify potential records to map the use of systematic review evidence for formulating NHPs under the MoHFW, Government of India. One of the prominent limitations of the paper is that the findings are based on available and documented information only. We did not involve any stakeholders or their feedback while searching and analysing the use of systematic reviews in formulating public health policies for NHPs in India. This might have limited our perceptions of the usefulness of systematic reviews for policy decisions and key contextual influences on evidence use for policymaking. Secondly, the search was restricted to the English language, this might have led to non-inclusion of some potential records as India is a linguistically diverse country with each State of the country publishing their reports in regional languages/official language of the states. The authors have not attempted to search state-level or state-specific departments and ministry websites to identify recommendations, guidelines and any other potential records. Nevertheless, the state-specific guidelines are framed based on the national guidelines, therefore, we anticipate that we might not have missed important documents. Neither did we search specifically for citations of WHO guidance in Indian policy documents, which could inform policies indirectly with systematic review evidence.

### Recommendations

This document analysis shows that India has used systematic review evidence for some of its high-priority programmes and is increasingly using evidence synthesis for developing guidelines. Through this paper we recommend increased visibility of evidence in all NHPs as is seen in NTEP, NACP, NVBDCP, NCPDCS, UIP and RMNCH + A. Furthermore, there were a few NHPs that mentioned the use of systematic reviews without proper citation. There is a possibility that these NHPs have used systematic reviews but have not documented it. Better citation practice would increase the visibility of science and model the use of evidence in policy development.

We found that Indian data were often included in systematic reviews cited in the guideline documents; more often providing evidence about the extent of policy problems than systematic reviews assessing policy options. This finding emphasizes the need for methodologies that transfer global evidence about policy options to local settings. This is required if international guideline development organizations (for example, the WHO), are to highlight or flag the local region-specific (specifically LMICs) considerations such as benefits, cost, context-specific considerations, while developing international guidelines for global public health related problems. Collaboration between international guidelines development organizations and regional or national institutions (e.g. with remits for India) could enhance transferability of evidence and provide contextual insights for transferring or adapting international guidelines to the Indian context.

Additionally, given the lack of qualitative systematic reviews informing the policy documents analysed, we recommend that policy development groups broaden their interest in systematic reviews to consider qualitative research that includes global evidence relevant to issues that are priorities for India. For instance, there are many systematic reviews of qualitative research which, together, offer in-depth understanding of contextual factors that influence women’s choice and use of contraception (D’Souza *et al.*, 2022). Similarly, a synthesis of qualitative evidence of recipient and provider views has shed light on implementation challenges encountered with rapid molecular tests for TB and TB drug resistance (Engel *et al.*, 2022).

## Conclusion

India is increasingly contributing to, and explicitly making use of, global efforts to systematically review health research to make policy decisions in health, mainly in the areas of perinatal health and communicable diseases. Over the years, India has successfully used systematic review evidence for some of its high-priority NHPs and has documented the same in national guidelines. Some documents supporting implementation of policy decisions also cite systematic reviews, although more to highlight the need for action and justify policy decisions than to guide implementation. The visibility of systematic review evidence and understanding about the use of evidence in NHPs will shape the future of guideline development and public health policy making in India.

## Data Availability

All data relevant to the study are included in the article. The dataset will be available on request from the corresponding author.

## Abbreviations

AIDS = Acquired immuno-deficiency syndrome,

EBM = evidence-based medicine,

HIV = human immunodeficiency virus,

HTAIn = Health Technology Assessment in India,

INCLEN = International Clinical Epidemiology Network,

KMC = kangaroo mother care,

KTA = knowledge to action,

LMIC = low- and middle-income countries,

MoHFW = Ministry of Health and Family Welfare,

NHP = National Health Programmes,

NACP = National AIDS Control Programme,

NMHP = National Mental Health Programme,

NPCDCS = National Programme for Prevention and Control of Cancer, Diabetes, Cardiovascular diseases and Stroke,

NTEP = National Tuberculosis Elimination Programme,

NVBDCP = National Vector Borne Disease Control Programme,

RCH = Reproductive and Child Health,

RMNCH+A = Reproductive, Maternal, Newborn, Child and Adolescent Health,

RNTCP = Revised National Tuberculosis Control Programme,

STD = sexually transmitted diseases,

TB = tuberculosis,

UIP = Universal Immunization Programme.

## Reflexivity statement

The inclusivity and balance within the authors’ groups is maintained as the authors of this paper represent both global North and global South regions. Additionally, the authors’ group is a mix of experienced senior academicians, research scholars and early career researchers. The interdisciplinary team of authors representing different subject backgrounds such as Public Health, Nursing, Social Sciences, Evidence Synthesis and Public Policy also adds to the inclusivity and balance.
